# The Role of *REL* and *IL2* and Their Polymorphisms in the Pathogenesis of Vitiligo—Exploratory Genetic Association Study

**DOI:** 10.3390/ijms27115084

**Published:** 2026-06-04

**Authors:** Marcelina Kądziela, Monika Migdalska-Sęk, Daria Domańska-Senderowska, Magdalena Kutwin, Ewa Brzeziańska-Lasota, Anna Woźniacka

**Affiliations:** 1 Department of Dermatology and Venereology, Medical University of Lodz, Pl. Hallera 1, 90-647 Lodz, Poland; magdalena.kutwin@umed.lodz.pl (M.K.); anna.wozniacka@umed.lodz.pl (A.W.); 2 Department of Biomedicine and Genetics, Biology and Medical Microbiology, Medical University of Lodz, 92-215 Lodz, Poland; daria.domanska-senderowska@umed.lodz.pl (D.D.-S.); ewa.brzezianska@umed.lodz.pl (E.B.-L.)

**Keywords:** vitiligo, *REL* gene, *IL2* gene, genetic polymorphism, rs2069763, rs6545836, gene expression, autoimmune disease, NF-κB pathway, PBMCs

## Abstract

Vitiligo is a chronic autoimmune skin disorder characterized by progressive melanocyte loss and depigmented skin lesions, whose pathogenesis has been attributed to genetic and immunological factors. The aim of this exploratory study was to assess *REL* and *IL2* gene expression and evaluate the relationship between the presence of their polymorphisms (rs6545836 and rs2069763) and susceptibility to vitiligo and disease symptoms. The study included 100 patients with vitiligo and 47 healthy controls matched for age and sex. Gene expression in peripheral blood mononuclear cells was assessed using real-time PCR and the ΔΔCT method. Higher *REL* expression was noted in patients compared with controls (median RQ = 1.52 vs. 1.22), although the difference was not significant (*p* = 0.919). *IL2* gene expression remained below the detection threshold in all analyzed samples. Significant differences in *IL2* rs2069763 distribution was found between patients and controls (*p* = 0.0059, χ^2^ = 7.57, chi-square test; *p*_adj = 0.02636, Bonferroni correction), with the C allele being more frequent among vitiligo patients and the A allele showing a potential protective effect. Although the CC genotype was initially associated with increased disease risk, this association did not remain significant after correction for multiple comparisons. No significant association between *REL* rs6545836 and overall susceptibility to vitiligo was identified. Nevertheless, rs6545836 was associated with *REL* expression levels (Kruskal–Wallis test, *p* = 0.0032; *p*_adj = 0.0096, Bonferroni correction). While the overall analysis indicated associations between *REL* rs6545836 and clinical severity indices (BSA, VES, and VASI), most subgroup associations lost significance after Bonferroni correction; the VASI only demonstrated an insignificant relationship (*p*_adj = 0.0729). The obtained findings suggest that *IL2*- and *REL*-related immune pathways influence vitiligo pathogenesis and clinical heterogeneity; however, these exploratory findings should be interpreted cautiously and require confirmation in studies with greater statistical power.

## 1. Introduction

Vitiligo is a skin disease characterized by depigmented patches of various sizes in different locations and a heterogeneous course. The etiopathogenesis of this disease remains unclear. While several hypotheses have been proposed, the consensus is that autoimmune processes play an important role [1,2]. Indeed, patients with vitiligo frequently exhibit autoimmune diseases, such as Hashimoto’s disease, Graves’ disease, alopecia areata, type 1 diabetes, rheumatoid arthritis, systemic lupus erythematosus (SLE), systemic sclerosis (SSc), psoriasis, and pernicious anemia [3,4,5]. Currently, not all risk factors or factors for the development of vitiligo have been identified, which are associated with an increased risk of co-occurrence of autoimmune diseases or are correlated with disease activity and severity. The condition has demonstrated high heritability and polygenic transmission associated with the activity of a number of risk alleles, each of which has little effect on its own [6,7]. Several candidate genes and genetically linked loci have been identified that may influence susceptibility to both generalized vitiligo and other autoimmune diseases.

A recent study found that the *REL* and *IL2* genes may be new susceptibility candidates [8]. In particular, it has been proposed that certain single-nucleotide polymorphisms (SNPs), including *REL* rs6545836 and *IL2* rs2069763, may influence individual susceptibility to vitiligo and its clinical phenotype by modulating gene expression and the immune response [8]. Both genes are known to be involved in regulatory pathways linked to other autoimmune diseases. *IL2* is associated with signaling and promoting the growth and proliferation of T cells and NK cells and regulatory T cell (Treg) development [9]. It also plays a crucial role in initiating the immune response. *REL*, on the other hand, activates various pathways, such as NF-κB, contributing to immune function. *C-REL* is expressed in hematopoietic cells and regulates T-cell development and antigen presentation by influencing the transcription of various genes related to adaptive immunity [10].

The aim of the present study was to determine the role of *IL2* and *REL* in the development of vitiligo. The results were correlated with clinical data and selected indicators of the activity and severity of the disease: BSA (Body Surface Area), VIDA (Vitiligo Disease Activity Score), VES (Vitiligo Extent Score) and VASI (Vitiligo Area Scoring Index). The study is one of the first to evaluate the expression of *IL2* and *REL* and their polymorphisms in the context of vitiligo and to correlate the findings with clinical measures of disease activity and severity.

## 2. Results

### 2.1. Relative Expression of REL and IL2

The relative expression (RQ) of *REL* and *IL2* was assessed in a group of patients with vitiligo and compared to the control group. *REL* mRNAs were expressed in all evaluated samples. Increased expression compared to the calibrator (RQ > 1) was observed in 72% of the vitiligo group samples and 74.5% of the control group. Higher *REL* mRNA expression was noticed in the vitiligo group versus the control (median RQ: 1.523 and 1.219, respectively), but this difference was statistically insignificant (*p* = 0.9192; Mann–Whitney U-test). The RQ values of the *IL2* gene were below the detection threshold of the qPCR assay (no amplification detected/Ct indeterminate). The results are shown in Table 1.

The relative expression of the *REL* gene was compared with the clinical features of patients with vitiligo. No significant differences in gene expression levels were found between patients divided by BMI (<25; Median RQ = 1.566 vs. >25; Median RQ = 1.506) or by smoking status (smokers; Median RQ = 1.225 vs. non-smokers; Median RQ = 1.610) (*p* = 0.590 and *p* = 0.298, respectively; Mann–Whitney U test). Regarding time of disease onset, no significant differences were observed between patients with early (Median RQ = 1.487) and late onset of disease (Median RQ = 1.613); (*p* = 0.551, Mann–Whitney U test).

Similarly, no significant differences in *REL* expression were found between patients with co-occurring autoimmune diseases (median RQ = 1.674) and patients without such conditions (median RQ = 1.441); (*p* = 0.585, Mann–Whitney U test). Also, no significant difference in *REL* expression were found between patients with a family history of vitiligo (Median RQ = 1.484) and those without (Median RQ = 1.611); (*p* = 0.388, Mann–Whitney U test). However, *REL* demonstrated a higher RQ value in patients with a family history of autoimmune diseases (Median RQ = 1.743) compared to those without (Median RQ = 1.399), but these differences were insignificant (*p* = 0.131, Mann–Whitney U test).

Regarding the clinical course of the disease, no significant differences in *REL* expression were found between patients with stable (Median RQ = 1.441), progressive (Median RQ = 1.610), rapidly progressive (Median RQ = 1.980), and regressive courses (Median RQ = 1.677), (*p* = 0.791, Kruskal–Wallis test). For vitiligo type, the highest expression was observed in unclassified vitiligo (median RQ = 2.042), followed by non-segmental vitiligo (median RQ = 1.522) and then segmental vitiligo (median RQ = 1.083); however, the differences were not statistically significant (*p* = 0.581, Kruskal–Wallis test). Likewise, *REL* expression did not differ significantly between clinical subtypes of vitiligo: generalized (Median RQ = 1.506), generalized mucosal (Median RQ = 2.264), focal (Median RQ = 2.968), acrofacial (Median RQ = 1.371, and universal (Median RQ = 1.165); *p* = 0.198 (Kruskal–Wallis test). Finally, no significant differences in *REL* expression were found with regard to selected clinical indicators used to assess the severity and activity of the disease and the extent of skin lesions (BSA, VIDA, VES, and VASI).

### 2.2. Distribution of Alleles and Genotype Frequencies of rs6545836 (REL) and rs2069763 (IL2)

Polymorphisms rs6545836 in the REL gene and rs2069763 in the IL2 gene were identified in the group of patients with vitiligo and in the control group. For each SNP, the frequencies of alleles A and G and A and C, respectively, were determined. At the rs6545836 polymorphic site, genotypes AA, AG, and GG were identified, with respective genotype counts of 6, 59, and 35 in the patient group and 5, 21, and 21 in the control group. A representative allele discrimination plot for rs6545836 is shown in Supplementary Figure S1. At the rs2069763 polymorphic site, the identified genotypes were AA, AC, and CC, with respective genotype counts of 19, 51, and 30 in the patient group and 16, 26, and 5 in the control group. The obtained data were consistent with Hardy–Weinberg equilibrium, except for rs6545836 in the patient group, where deviation from HWE was observed (χ^2^ goodness-of-fit test). The results are presented in Table 2.

Allele frequencies (A and C for rs2069763; A and G for rs6545836) were compared between patients and controls. For rs2069763, significant differences in the distribution of alleles A and C were found between the studied groups (*p* = 0.0059, χ^2^ = 7.57, chi-square test; *p*_adj = 0.02636, Bonferroni correction). In contrast, for rs6545836, no significant differences were observed in the distribution of alleles A and G (*p* = 0.6719, χ^2^ = 0.18, chi-square test; *p*_adj = 1.0, Bonferroni correction).

Genotype frequencies of rs6545836 and rs2069763 were also compared between the patient and control groups. For rs2069763, significant differences in the distribution of genotypes AA, AC, and CC were demonstrated between the groups (*p* = 0.0167, χ^2^ = 8.1865, chi-square test of independence); however, they did not remain significant after Bonferroni correction (*p*_adj = 0.0668). For rs6545836, no significant differences in the distribution of genotypes AA, AG, and GG were observed between patients and healthy controls (*p* = 0.2334, χ^2^ = 2.9104, chi-square test of independence; *p*_adj = 0.9336, Bonferroni correction).

In order to identify the alleles and genotypes (rs6545836 and rs2069763) associated with significant differences in the risk of vitiligo, odds ratios (ORs) with 95% confidence intervals (95% CIs) were calculated between the study and control group. The obtained results indicate that the CC genotype of rs2069763 was significantly associated with an increased risk of vitiligo in the uncorrected analysis (OR = 3.60; *p* = 0.0181); however, this association did not remain significant after Bonferroni correction (*p*_adj = 0.0724). A significant association was observed between the C allele of rs2069763 and the manifestation of disease (OR = 2.009; *p* = 0.0059; *p*_adj = 0.0236, Bonferroni correction). No significant association between the other SNP (rs6545836) and disease was observed in either genotype- or allele-based analyses. The OR values for the analyzed genotypes of the studied polymorphisms between the two groups are presented in Table 3.

The relationships between the genotypes of the rs6545836 and rs2069763 variants were compared with the clinical features of the vitiligo patients. No significant difference in either polymorphism was observed with regard to the time of disease onset (*p* = 0.8530 and *p* = 0.1651; respectively, Kruskal–Wallis test) nor the duration of the disease (*p* = 0.0923 and *p* = 0.7706; respectively, Kruskal–Wallis test). In the study group, no significant differences in individual rs6545836 and rs2069763 genotypes were found with regard to BMI value (*p* = 0.8532 and *p* = 0.4326, Kruskal–Wallis test).

**Figure 1:**
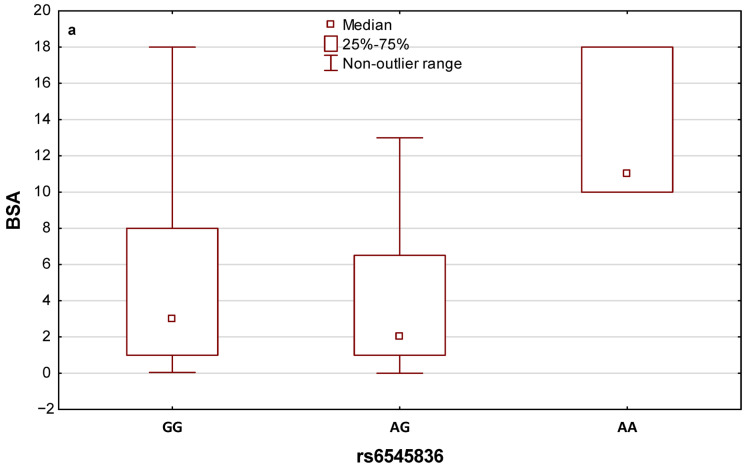

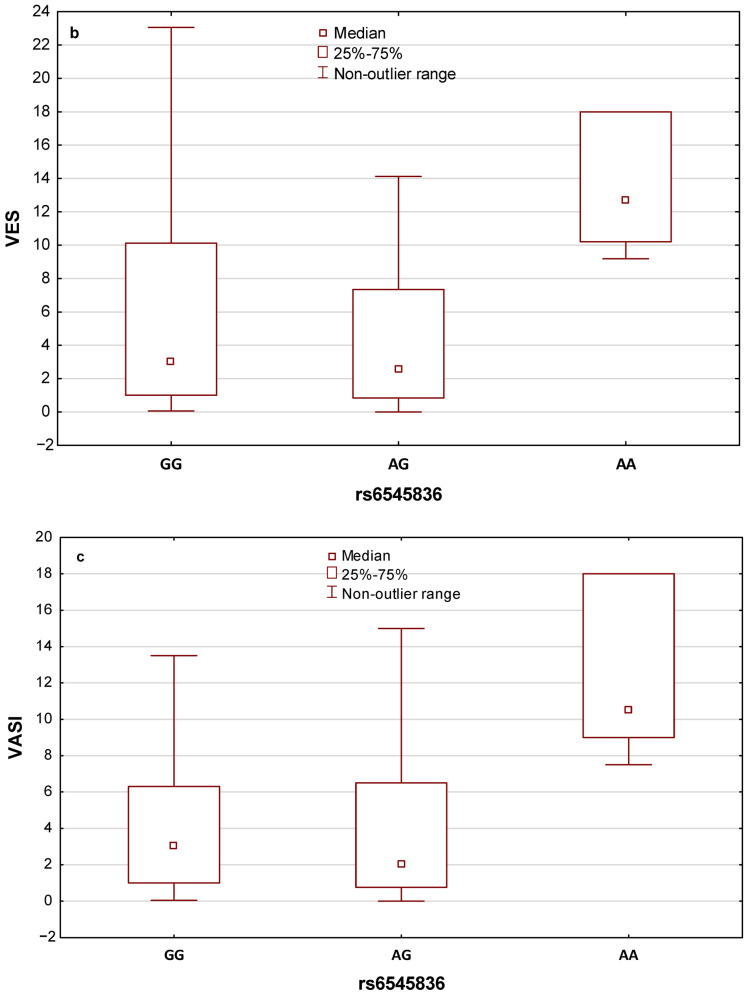
Box-and-whisker plot showing levels of (**a**) BSA, (**b**) VES, (**c**) VASI depending on rs6545836 genotypes in patients with vitiligo.

A significant association was found between the rs6545836 polymorphism and clinical indices used to assessment disease severity and disease activity (BSA, VES, and VASI) (Kruskal–Wallis test: BSA, *p* = 0.0191; VES, *p* = 0.0195; and VASI, *p* = 0.0148). Significant differences in the VES and VASI were noted between GG and AA (*p* = 0.0481 and *p* = 0.0488, respectively; Newman–Keuls *post hoc* test); however, after adjustment for multiple comparisons, these differences were not significant (*p*_adj = 0.1443 and *p*_adj = 0.1464; respectively). Similarly, the *post hoc* analysis initially suggested that the VASI differed significantly between AA vs. AG (*p* = 0.0243), but these associations lost their significance after Bonferroni correction (*p*_adj = 0.0729). The BSA index was slightly higher for AA patients compared to GG (*p* = 0.0525, Newman–Keuls *post hoc* test), but this relationship lost significance after correction (*p*_adj = 0.1575). The results are presented in Figure 1.

### 2.3. Association of rs6545836 Genotype with REL Expression Level

**Figure 2:**
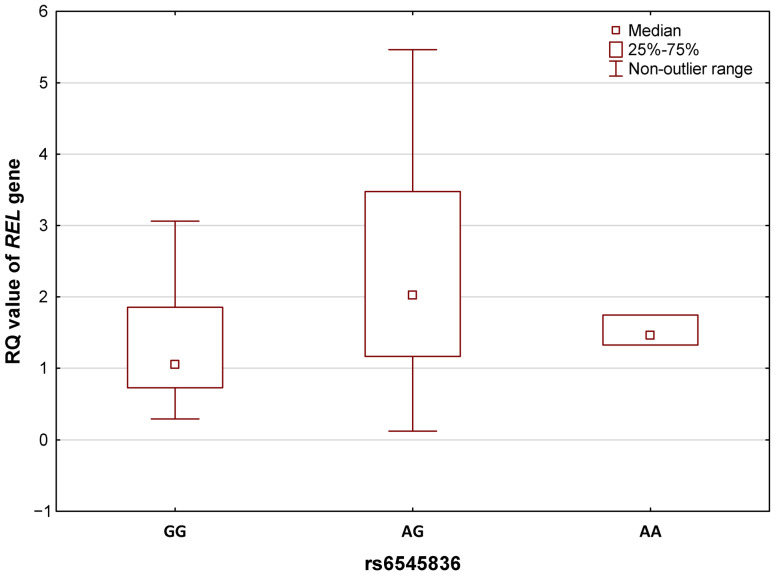
Box-and-whisker plot showing the level of *REL* expression depending on the rs6545836 genotype in patients with vitiligo.

The relationship between the rs6545836 genotype and *REL* gene expression was evaluated in both the vitiligo and control groups. Significant differences in *REL* gene expression levels were observed among patients with vitiligo depending on the genotype (Kruskal–Wallis test, *p* = 0.0032; *p*_adj = 0.0096, Bonferroni correction). The highest *REL* expression was associated with AG (Median RQ = 2.0196) and the lowest expression level with GG (Median RQ = 1.0500). The Newman–Keuls *post hoc* test confirmed significant differences in *REL* expression between GG vs. AA (Newman–Keuls test, *p* = 0.000078; *p*_adj = 0.000234, Bonferroni correction) and between AG vs. AA (Newman–Keuls test, *p* = 0.000094; *p*_adj = 0.000282, Bonferroni correction). The results are shown in Figure 2. In the control group, no statistically significant association was found between the rs6545836 genotype and *REL* gene expression.

## 3. Discussion

Vitiligo is a complex, multifactorial skin disorder characterized by the progressive loss of melanocytes, leading to depigmented patches on the skin. Although the exact pathogenesis remains unclear, accumulating evidence suggests that autoimmune mechanisms play a dominant role in melanocyte destruction [3,11]. The present study focused on two genes with known roles in immune regulation: *REL* and *IL2*. The selected polymorphisms, and their genes, have also demonstrated susceptibility to various autoimmune diseases, including vitiligo, in previous genetic studies [12].

Our findings indicate that the predominant subtype in the patient group was non-segmental vitiligo (93%). This is consistent with previous data reporting that non-segmental forms account for the vast majority of cases worldwide [13,14]. The mean age at disease onset was 29 years, with 43% of patients experiencing early onset before age 25. This distribution is in line with previous reports suggesting that almost half of patients with vitiligo present before the age of 20. Interestingly, a significant proportion of patients (57%) presented with late-onset vitiligo, emphasizing the heterogeneity of the disease regarding age at presentation. A previous literature review found the prevalence of late-onset vitiligo to range from 6.5% to 14.7%, with an age cut-off of 30 years [15]. One study suggests that late-onset vitiligo is associated with a higher risk of nonautoimmune diseases, positive family history of vitiligo, and leukotrichia [16].

Our patients exhibited a high prevalence of comorbid autoimmune or atopic disorders (58%), which supports the strong immunological basis of vitiligo. The most frequent autoimmune disorders in patients with vitiligo are type 1 diabetes, rheumatoid arthritis, systemic lupus erythematosus (SLE), autoimmune thyroiditis, Addison’s disease, alopecia areata, psoriasis, and systemic sclerosis (SSc) [8,13,15]. A ten-year retrospective study of 3280 patients identified comorbid autoimmune conditions in 23%, among whom the most common conditions were thyroid disorders and psoriasis [16]. A study of 1098 patients with vitiligo found nearly 20% to have at least one comorbid autoimmune disease [5]. A considerable proportion of our patient group reported a positive family history of autoimmune diseases, suggesting the involvement of shared genetic or immunological pathways. Large-scale genetic and epidemiological studies based on European populations have found the prevalence of vitiligo in first-degree relatives of probands to be 6%, supporting a polygenic, multifactorial mode of inheritance as well as an age-related onset of vitiligo [17]. However, the concordance rate of vitiligo in monozygotic twins was only 23%, suggesting that environmental or other non-genetic factors also play a significant role in its pathogenesis [17].

### 3.1. REL Gene Expression

Higher median *REL* expression was noted in vitiligo patients compared to controls, although the difference was not significant. *REL* encodes a subunit of Nuclear factor κB (NF-κB), a central regulator of immune and inflammatory responses. NF-κB comprises a family of structurally related transcription factors, which include RelA, RelB, c-Rel, NF-κB1, and NF-κB2 [18]. The NF-κB pathway plays a crucial role in the activation, survival, and differentiation of T cells, including Th1 and Th17 subsets, as well as in the maintenance of regulatory T cells [19]. Dysregulation of NF-κB has been implicated in autoimmune diseases such as rheumatoid arthritis, lupus, psoriasis and multiple sclerosis [20,21]. The c-Rel protein is primarily expressed in inflammatory cells, and although mice lacking c-Rel do not develop infectious diseases, they exhibit impaired Th17 cell-mediated immune responses [22].

In vitiligo, NF-κB activation may contribute to melanocyte destruction by enhancing the release of pro-inflammatory cytokines and promoting autoreactive T-cell survival [19]. NF-κB-dependent upregulation of pro-inflammatory cytokines and chemokines promotes the recruitment and activation of autoreactive CD8^+^ T cells that target melanocytes; this would be consistent with the IFN-γ–CXCL9/CXCL10-driven model of disease [23].

Vitiligo lesions have been found to demonstrate elevated levels of hydrogen peroxide (H_2_O_2_) and the pro-inflammatory cytokine interleukin-6 (IL-6), a critical mediator in autoimmune disease development [24]. This suggests upregulation of IL-6 in melanocytes induced by H_2_O_2_ through the p38 and NF-κB signaling pathways, could represent a molecular link between oxidative stress and inflammatory or autoimmune responses in vitiligo. This may offer a potential therapeutic target for disease treatment [24].

Of note, patients with a family history of autoimmune diseases tended to exhibit higher *REL* expression compared to those without. Although not statistically significant, this observation may suggest the possible involvement of NF-κB-related pathways, as previously suggested in studies on systemic autoimmune conditions [25].

The study also assessed *REL* gene expression relative to disease activity and extent indices, including VASI, VES, and BSA. While no statistically significant associations were identified, non-significant increases in *REL* expression were observed in patients with moderate disease activity. This suggests that NF-κB signaling may play a modulatory role in shaping disease severity; however, due to the clinical heterogeneity of the disease, it is difficult to establish significant correlations between molecular markers and vitiligo extent [26].

### 3.2. IL2 Gene Expression

Unfortunately, no reliable assessment of *IL2* transcriptional activity was possible as *IL2* gene expression was below the detection threshold in all analyzed samples. As a result, the conclusions regarding *IL2* are based mainly on genetic association data rather than direct evidence of functional expression. IL2 is a pivotal cytokine in immune regulation, responsible for T-cell proliferation, survival, and maintenance of Tregs [27]. In the adult lymphoid compartment, peripheral T-reg cells proliferate at a substantially higher homeostatic rate than conventional naive or memory T cells to compensate for their high turnover rate; the cells are also needed to sense autoantigens and IL-2. Consequently, intact IL-2/IL-2R signaling is crucial for Treg maintenance [28].

Dysregulation of IL2 production has been implicated in several autoimmune diseases, including type 1 diabetes and systemic lupus erythematosus [29]. In vitiligo, elevated serum IL2 or soluble IL2 receptor levels have been reported, indicating heightened immune activation [30,31]. Some authors have also noted a negative correlation between IL-2 level and duration of disease and a positive correlation with activity. Several studies have demonstrated elevated levels of the soluble interleukin-2 receptor (sIL-2R) in the plasma of patients with vitiligo, as well as in tissue biopsy specimens obtained from vitiligo lesions. However, only one study appears to have evaluated *IL2* mRNA expression in vitiligo, and the findings did not reveal any significant difference between patients and controls. This lack of detection does not exclude the pathogenic relevance of IL2; however, it does highlight the importance of studying activated immune cells or in situ expression within lesional skin.

### 3.3. rs2069763 Polymorphism of the IL2 Gene

A significant association was observed between the rs2069763 polymorphism of the *IL2* gene and the risk of developing vitiligo. The CC genotype was associated with approximately a threefold increased risk of the disease compared with the reference genotype; however, this effect did not remain significant after Bonferroni correction. As such, it should be interpreted as a trend, indicating that the variant may have a possible predisposing role. Similarly, the presence of the C allele significantly increased the risk of vitiligo, while the A allele may have a protective effect, indicated by a lower frequency among patients with vitiligo. These findings may suggest a possible role for the rs2069763 polymorphism in vitiligo susceptibility, further supporting the concept of a genetic contribution to this immune-mediated disease. Martins et al. also report a possible association between the *IL2* marker rs2069763 and vitiligo, particularly in patients with late-onset disease, concomitant autoimmune disorders, and the non-segmental subtype [8].

Although similar trends were observed in both genotypic and allelic analyses, these findings should be interpreted cautiously and require validation in larger independent cohorts before rs2069763 can be considered a reliable genetic marker of disease susceptibility [8]. Interestingly, rs2069763 has also been implicated in other autoimmune diseases, including juvenile systemic lupus erythematosus, autoimmune hepatitis, new-onset diabetes after transplantation, multiple sclerosis, and rheumatoid arthritis [18,30].

### 3.4. rs6545836 Polymorphism of the REL Gene

A significant association was found between the rs6545836 polymorphism and the level of *REL* gene expression, suggesting that the SNP may have functional relevance in disease pathogenesis; it is possible that rs6545836 may modulate the transcriptional activity of *REL* and thereby influence the regulation of the immune response. Significant differences in *REL* expression were observed between the GG vs. AA and AG vs. AA genotypes, which further confirm that specific allelic variants are associated with altered REL transcript levels. These findings suggest that a particular allelic configuration may determine activation of the NF-κB signaling pathway, with *REL* playing a key role as a transcription factor regulating inflammatory responses and autoimmune processes [18,31].

The AA genotype of the *REL* rs6545836 polymorphism was associated with greater clinical severity of vitiligo, as reflected by higher BSA, VASI, and VES scores compared with AG and GG genotypes. However, these associations did not remain statistically significant after Bonferroni correction, with only a trend observed for VASI scores. Thus, while patients with the AA genotype consistently showed higher severity indices, suggesting that this variant may predispose to a more severe clinical course of vitiligo, and it may modulate the clinical phenotype of the disease, these results should be interpreted with caution. While rs6545836 may have a possible effect on disease severity, our findings but require confirmation and validation in larger, independent cohorts and ideally in functional studies. A similar study, conducted in a family-based cohort and an independent case–control sample, demonstrated a significant association between *REL* variants and patients with early disease onset, non-segmental vitiligo, and concomitant autoimmune comorbidities [8].

Although allele frequencies were generally comparable to European population-based datasets, rs6545836 was found to deviate from the Hardy–Weinberg equilibrium in the patient group; this may reflect potential artifacts, limited sample size, or a true biological association. In contrast, no such deviation was observed for the control group, as indicated by quality control procedures and genotype clustering inspection performed for the entire dataset. However, as independent replication of genotyping was not possible, the results of the genetic association analysis should be interpreted with caution and considered preliminary. Again, further confirmation and validation is needed in larger, independent cohorts with more precise population stratification.

The study has some limitations. The first is the relatively small size of the control group. This imbalance may reduce the statistical power to detect modest genetic effects and increase the risk of both type I and type II errors. Also, the subgroup analyses used a large number of statistical tests, including comparisons across clinical characteristics, disease severity indices, and genotype–expression relationships. To reduce the risk of type I error, the main association analyses included the Bonferroni correction for multiple comparisons. Furthermore, gene expression was assessed only in PBMCs. Although PBMCs provide valuable information on systemic immune alterations, they may not fully capture the local immune and inflammatory processes occurring within vitiligo lesions. Finally, the *IL2* mRNA level in PBMC was below the detection threshold of the qPCR assay; as such, our conclusions regarding the potential function of *IL2* refer only to the genetic associations observed in the studied cohort, based on previous literature, rather than to directly confirmed functional mechanisms. Therefore, these findings should be interpreted with caution, and further studies using alternative experimental models and more sensitive methods for assessing *IL2* expression are needed.

Our findings confirm the clinical heterogeneity of vitiligo and its frequent coexistence with autoimmune and atopic diseases. However, it does not allow for definitive conclusions regarding the role of *IL2* gene expression in disease pathogenesis. The lack of detectable *IL2* mRNA expression in resting PBMCs suggests that further studies using activated lymphocyte models may be necessary to adequately assess its transcriptional activity. Moreover, its potential local role in cutaneous immune dysregulation in vitiligo may be clarified by evaluation of *IL2* expression directly within lesional skin biopsies. The presence of the rs2069763 polymorphism within the *IL2* gene was associated with altered allele distribution between patients and controls, indicating a possible contribution to disease susceptibility, with the C allele potentially increasing risk and the A allele exerting a protective effect; however, these associations did not remain significant after correction for multiple testing and should therefore be interpreted with caution. In turn, the presence of rs6545836 in *REL* was not associated with overall susceptibility to vitiligo at the genotype or allele frequency level. Nevertheless, this variant was linked to variability in *REL* expression, which was slightly increased in patients compared to controls. With respect to selected indicators of clinical severity, the observed trend of VASI changes depending on the rs6545836 genotype suggests a possible role in the modulation of the disease phenotype, rather than in its onset.

**Figure 3:**
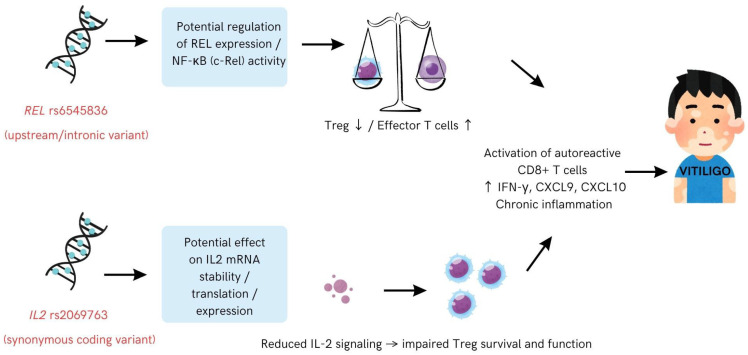
Hypothetical immunogenetic model linking *REL* and *IL2* polymorphisms with immune dysregulation in vitiligo.

The *IL2* rs2069763 polymorphism is a single nucleotide variant located within the coding sequence and classified as a synonymous variant. This does not result in a change in the amino acid sequence of the IL2 protein, but it may potentially influence gene expression regulation, mRNA stability, or translational efficiency. In the context of vitiligo, rs2069763 may therefore be considered a potential marker of dysregulated *IL2* signaling, which plays an important role in T-cell activation, maintenance of Treg homeostasis, and preservation of immune tolerance. Interestingly, rs6545836 represents a non-coding variant located within the upstream and intronic regions of the *REL* gene, suggesting a potential regulatory rather than structural effect. It may be hypothesized that rs6545836 could modulate *REL* expression or NF-κB/c-Rel-dependent immune responses. Given the central role of c-Rel in T-cell activation and immune tolerance, this type of dysregulation could potentially contribute to impaired autoimmune regulation involved in melanocyte destruction in vitiligo. A summary of this hypothesis is illustrated in Figure 3.

## 4. Materials and Methods

### 4.1. Clinical Features of Patients

This exploratory study included 100 vitiligo patients, 56 women (56%) and 44 men (44%), aged from 18 to 76 years (mean age ± SD: 46.6 ± 16.39 years). The control group comprised 47 healthy individuals, 29 women (62%) and 18 men (38%), aged from 18 to 84 years (mean age ± SD: 39.8 ± 15.4) without family history of vitiligo. Exclusion criteria from participation in the study included pregnancy and lactation, use of anticoagulants, acute cardiovascular disorders, hematological disorders and cancers. Clinical evaluation of skin lesions included detailed physical examination. The scales BSA, VIDA, VES and VASI were used to assess disease activity and severity. The type of vitiligo was also assessed according to the modified classification from 2012 [32].

The study was conducted in accordance with the principles of Good Clinical Practice (GCP) and the guidelines of the Declaration of Helsinki. The study protocol was approved by the Bioethics Committee of the Medical University of Lodz (resolution no. RNN/49/23/KE). Patients completed a questionnaire with questions about demographics and medical history. Other data from patients were collected from medical reports: age, sex, height, weight, simulants, age of disease onset, distribution of vitiligo lesions, co-occurrence of other autoimmune diseases, occurrence of vitiligo and other autoimmune diseases in the family, location of skin lesions, and previous treatment.

The demographics of the group are presented in Table 4. The mean duration of vitiligo in the entire group was 17.56 ± 14.00 years. The mean age at disease onset was 29.02 ± 16.06 years. Early onset of the disease (before the age of 25) was recorded in 43 patients (43%), while late onset (after 25 years) was observed in 57 patients (57%). Coexisting autoimmune or atopic diseases were reported in 58 patients (58%). The most commonly observed comorbidities included allergic rhinitis (n = 18), Hashimoto’s thyroiditis (n = 15), and bronchial asthma (n = 12). A positive family history of vitiligo was noted in 23 individuals (23%). Additionally, 43 patients (43%) reported a family history of autoimmune diseases. Among the participants, 16 patients (16%) were smokers, while 84 (84%) were non-smokers. In the six months preceding the study, 21 patients (21%) had undergone some form of treatment (topical or phototherapy).

### 4.2. Blood Collection and Procedure for Obtaining PBMC

First, 5 mL of whole blood in EDTA was collected from each patient in order to isolate genetic material (DNA, RNA). The blood was centrifuged on a density gradient using Histopaque-1077 (Sigma-Aldrich, Poznań, Poland) according to the manufacturer’s protocol. The peripheral blood mononuclear cell (PBMC) pellet was divided into two aliquots. One portion was suspended in 1xPBS for DNA isolation; the other was suspended in RNA Fix for RNA isolation and frozen at −20 °C.

### 4.3. DNA Isolation, Qualitative and Quantitative Assessment of DNA, SNP Genotyping

SNP rs6545836 in *REL* and SNP rs2069763 in *IL2* were selected for study based on the supposed role of their genes in vitiligo. Total DNA was isolated from the lymphocyte sediment using the GeneMAGNET Human and Animal Tissue DNA Purification Kit (EURx Sp. z o. o., Gdansk, Poland) according to the manufacturer’s protocol. The isolated DNA was subjected to qualitative and quantitative assessment by measuring its absorbance at 260/280 nm using the Eppendorf BioPhotometerTM Plus device (Eppendorf, Hamburg, Germany). The prepared DNA was divided into portions and frozen at −80 °C.

SNP analysis was performed using TaqMan^®^ SNP Genotyping Assay probes, (Applied Biosystems, Carlsbad, CA, USA): C_2080999_10 (rs6545836) and C_15859920_10 (rs2069763), using real-time polymerase chain reaction (qPCR) assays. Real-time PCR was carried out on a 7900HT Fast Real-Time PCR System (Applied Biosystems, Carlsbad, CA, USA) to annotate the allele of each DNA sample. The reaction was performed using a 20 µL reaction mixture: 10.0 µL TaqMan Universal Master Mix (2×), 1.0 µL TaqMan^®^ SNP probe, 1.0 µL DNA template and 8.0 µL RNase-free water. The reaction was performed in a Gradient Mastercycler thermal cycler (Eppendorf, Hamburg, Germany) using the following cycle: initial denaturation at 95 °C for 10 min, followed by denaturation for 40 cycles at 95 °C for 30 s and an annealing/extension step at 60 °C for one min.

Fluorescence intensity was measured in the wells of the Taq Man plate, and the fluorescence data was evaluated using SDS 2.4 automatic allele calling software (Applied Biosystems, Carlsbad, CA, USA). Genotyping was performed in a single experimental run, according to the manufacturer’s protocol. Samples with an initially unassigned genotype, clearly outliers, or locations between the main clusters were subjected to additional quality control assessment before final genotype assignment. To minimize potential technical bias, repeat genotyping of missing data included randomly distributed samples from both the vitiligo and control groups; these analyses were performed on the same genotyping plate. The percentage of missing genotypes was comparable between the treatment (16%) and control (13%) groups. A representative allele discrimination plot for rs6545836 is included in the Supplementary Materials.

### 4.4. RNA Isolation, Qualitative and Quantitative Assessment of RNA, Gene Expression Analysis

Total RNA was isolated from the lymphocyte sediment using the GeneMAGNET RNA Purification Kit (EURx Sp. z o. o., Gdansk, Poland) according to the manufacturer’s protocol. The isolated RNA was assessed qualitatively and quantitatively using a BioPhotometerTM Plus spectrophotometer (Eppendorf, Hamburg, Germany) at 260/280 nm. The prepared RNA was divided into aliquots and frozen at −80 °C.

The RNA was subjected to reverse transcription (RT) using a High-Capacity cDNA Reverse Transcription Kit (Applied Biosystems, Carlsbad, CA, USA) in a 20 µL volume: 10 x RT buffer, 25 x dNTP Mix (100 mM), 10 x RT Random Primers, MultiScribeTM Reverse Transcriptase, RNase Inhibitor and nuclease-free water. A total of 100 ng of total RNA was added to this. A negative control was prepared using water instead of RNA. The following RT reaction conditions were used: 10 min at 25 °C, 120 min at 37 °C, 5 min at 85 °C and cooling at 4 °C.

Relative gene expression was assessed by qPCR using a 7900HT Fast Real-Time PCR System (Applied Biosystems, Carlsbad, CA, USA). A total reaction volume of 20 µL was used containing the following: cDNA (1–100 ng), KAPA PROBE FAST suitable for qPCR (Kapa Biosystems, Cape Town, South Africa), RNase-free water, and 20xTaqMan^®^ Gene Expression Assay (Applied Biosystems, Carlsbad, CA, USA). The reaction targeted *REL* (Hs00968440_m1) and *IL2* (Hs00174114_m1), as well as *GAPDH* (Hs99999905_m1) as the reference gene. The relative expression of the tested genes was assessed using the delta-delta CT method (SDS 2.4 software, Applied Biosystems, Carlsbad, CA, USA) and presented as the RQ value relative to GAPDH. The calibrator RNA was isolated from a healthy patient without skin lesions.

### 4.5. Statistical Analysis

Statistical analysis was performed using Statistica 13.1 software (StatSoft, Cracow, Poland). The Shapiro–Wilk test showed that data were not normally distributed. The differences in the frequency of alleles and genotypes were assessed using the χ^2^ test. The compliance with Hardy–Weinberg equilibrium was assessed. The odds ratio (OR) was calculated with 95% confidence intervals (95% CI) to determine the strength and direction of the association between the individual genotypes and susceptibility to vitiligo. The Mann–Whitney U-test, Kruskal–Wallis test, or Neuman–Keuls’ multiple comparison test was used depending on the size of the groups. The results are shown as medians for all analyzed variables. Bonferroni correction (based on the total number of tests performed) was applied for multiple comparisons. Statistical significance was defined as *p* < 0.05.

## 5. Conclusions

Overall, our results support the hypothesis that immunoregulatory pathways involving *IL2* and *REL* may contribute to the pathogenesis of and clinical variability in vitiligo. However, the precise biological mechanisms behind them remain unclear. Due to the limited sample size and the exploratory nature of some analyses, further studies in larger and independent cohorts, combined with functional experiments, are needed to clarify the biological significance of these variants and their role in immune dysregulation in vitiligo.

## Figures and Tables

**Table 1:** Gene expression levels (median RQ values) and percentage of samples with decreased/increased levels in vitiligo patients and control group.

Samples	Vitiligo			Control		
		Sample Number (%)			Sample Number (%)	
Gene	RQ value	RQ < 1	RQ > 1	RQ value	RQ < 1	RQ > 1
*REL*	1.523	28 (28)	72 (72)	1.219	12 (25.5)	35 (74.5)
*IL2*	below detection level			below detection level		

**Table 2:** Frequencies of *REL* rs6545836 and *IL2* rs2069763 alleles and genotypes in the study and control group.

SNP	Group (n)	Allele (n)	Frequency	Genotype (n)	Frequency	HWE χ^2^ Goodness-of-Fit Test
rs6545836	vitiligo (100)	A (71)	0.355	AA (6)	0.060	χ^2^ = 8.3086 *p* = 0.0157
		G (129)	0.645	AG (59)	0.590	
				GG (35)	0.350	
	control (47)	A (31)	0.330	AA (5)	0.106	χ^2^ = 0.0056 *p* = 0.9972
		G (63)	0.670	AG (21)	0.447	
				GG (21)	0.447	
rs2069763	vitiligo (100)	A (89)	0.445	AA (19)	0.19	χ^2^ = 0.1049 *p* = 0.9489
		C (111)	0.555	AC (51)	0.51	
				CC (30)	0.30	
	control (47)	A (58)	0.617	AA (16)	0.341	χ^2^ = 1.3649 *p* = 0.5054
		C (36)	0.383	AC (26)	0.553	
				CC (5)	0.106	

**Table 3:** Odds ratios (ORs) and 95% confidence intervals (CIs) for alleles and genotypes of the rs6545836 and rs2069763 polymorphism in patients with vitiligo compared to the control group.

SNP	Genotype	OR	95% CI	*p* Value
rs6545836	AA	0.536	0.155–1.855	*p* = 0.5088, *p*_adj = 1.0; χ^2^ = 0.44; χ^2^ Yates analysis
	AG	1.782	0.885–3.587	*p* = 0.1040, *p*_adj = 0.4160; χ^2^ = 2.64; χ^2^ test
	GG	0.667	0.329–1.352	*p* = 0.2597, *p*_adj = 1.0; χ^2^ = 1.27; χ^2^ test
	A	1.192	0.709–2.006	*p* = 0.6719, *p*_adj = 1.0; χ^2^ = 0.18; χ^2^ test
	G	0.894	0.532–1.501	*p* = 0.6719, *p*_adj = 1.0; χ^2^ = 0.18; χ^2^ test
rs2069763	AA	0.454	0.208–0.995	*p* = 0.0458, *p*_adj = 0.1832; χ^2^ = 3.99; χ^2^ test
	AC	0.541	0.419–1.686	*p* = 0.6248, *p*_adj = 1.0; χ^2^ = 0.24; χ^2^ test
	CC	3.600	1.297–9.995	*p* = 0.0181, *p*_adj = 0.0724; χ^2^ = 5.58; χ^2^ Yates analysis
	A	0.498	0.302–0.821	*p* = 0.0059, *p*_adj = 0.0236; χ^2^ = 7.57; χ^2^ test
	C	2.009	1.218–3.315	*p* = 0.0059, *p*_adj = 0.0236; χ^2^ = 7.57; χ^2^ test

**Table 4:** Clinical and demographic characteristics.

	Patients: n = 100, (%)		Control (n = 47), (%)
Age	46.6 ± 16.39		39.8 ± 15.4
Sex	F = 56 (56)		F = 29 (62)
	M = 44 (44)		M = 18 (38)
BMI	<25 = 48 (48)		
	>25 = 52 (52)		
Type of vitiligo:			
Segmental type (uni-, bi- or plurisegmental)	3 (3)		
Nonsegmental (acrofacial, mucosal, generalized, universal, mixed)	93 (93)		
Nonclassified (focal, mucosal)	4 (4)		
VIDA	−1	11 (11)	
	0	38 (38)	
	1	44 (44)	
	2	5 (5)	
	3	1 (1)	
	4	1 (1)	
VASI	<1	23 (23)	
	1–1.9	20 (20)	
	2–5	25 (25)	
	6–19	21 (21)	
	20–100	11 (11)	
VES	<1	27 (27)	
	1.15–5	37 (37)	
	6–20	21 (21)	
	21–100	15 (15)	
BSA	0–1.5	35 (35)	
	2–5	29 (29)	
	6–19	21 (21)	
	20–100	15 (15)	
Course of the disease	Stable	39 (39)	
	Regression	12 (12)	
	Progressing	41 (41)	
	Rapidly progressing	8 (8)	

## Data Availability

The original contributions presented in this study are included in the article/Supplementary Material. Further inquiries can be directed to the corresponding authors.

## Supplementary Materials

The following supporting information can be downloaded at: https://www.mdpi.com/article/10.3390/ijms27115084/s1.

## Abbreviations

The following abbreviations are used in this manuscript:

AA	Homozygous genotype AA
AC	Heterozygous genotype AC
AG	Heterozygous genotype AG
BSA	Body Surface Area
BMI	Body Mass Index
CC	Homozygous genotype CC
cDNA	Complementary DNA
CI	Confidence Interval
DNA	Deoxyribonucleic Acid
EDTA	Ethylenediaminetetraacetic Acid
GCP	Good Clinical Practice
GG	Homozygous genotype GG
GAPDH	Glyceraldehyde-3-Phosphate Dehydrogenase
HWE	Hardy–Weinberg Equilibrium
IL2	Interleukin-2
NF-κB	Nuclear Factor Kappa B
NK cells	Natural Killer Cells
OR	Odds Ratio
PBMCs	Peripheral Blood Mononuclear Cells
PCR	Polymerase Chain Reaction
qPCR	Quantitative Polymerase Chain Reaction
RQ	Relative Quantification
SNP	Single-Nucleotide Polymorphism
Treg	Regulatory T cells
VES	Vitiligo Extent Score
VIDA	Vitiligo Disease Activity Score
VASI	Vitiligo Area Scoring Index
QV	quality value
