# Supplementary material for: The Role of REL and IL2 and Their Polymorphisms in the Pathogenesis of Vitiligo—Exploratory Genetic Association Study
Source: Int J Mol Sci. 2026 Jun 4;27(11):5084. doi: 10.3390/ijms27115084 (PMC13256598; doi:10.3390/ijms27115084)
Supplement: Supplementary file 1 [file ijms-27-05084-s001.zip › ijms-4246199-supplementary.pdf]

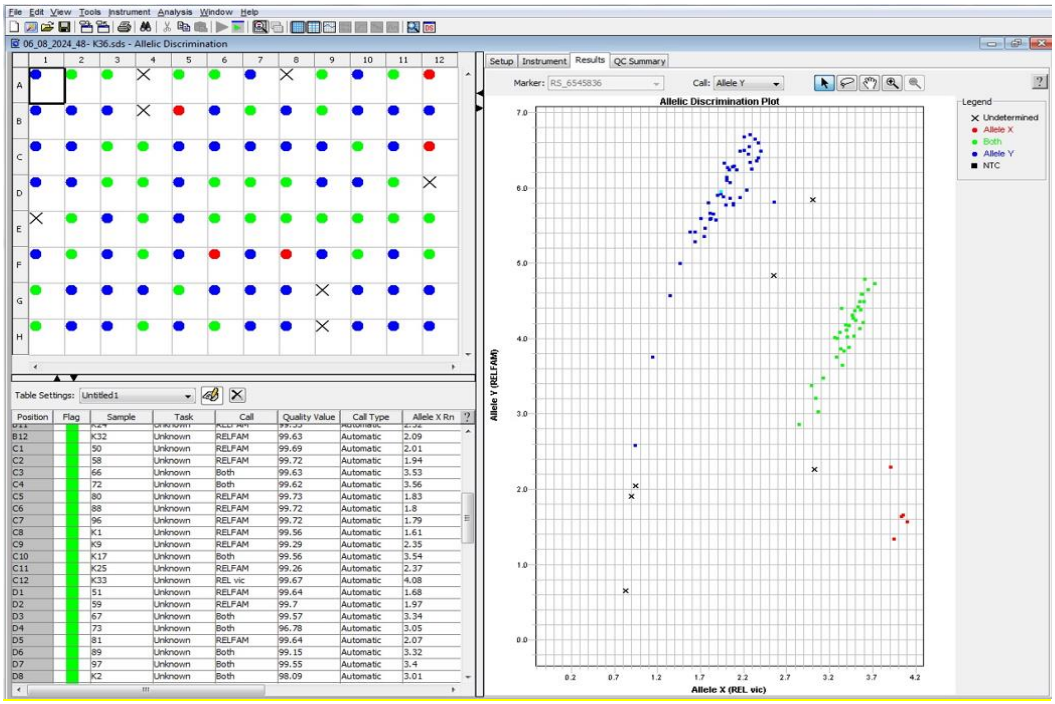

Figure S1. A representative allele discrimination plot for rs6545836 (high quality genotyping; QV>99%). Samples with ambiguous clustering or undetermined status were excluded during quality-control assessment, a subset of samples was re-genotyped.
